# A randomised controlled test of emotional attributes of a virtual coach within a virtual reality (VR) mental health treatment

**DOI:** 10.1038/s41598-023-38499-7

**Published:** 2023-07-17

**Authors:** Shu Wei, Daniel Freeman, Aitor Rovira

**Affiliations:** 1 Department of Psychiatry, University of Oxford, Warneford Hospital, Oxford, OX3 7JX UK; 2 Department of Experimental Psychology, University of Oxford, Oxford, UK; 3 Oxford Health NHS Foundation Trust, Oxford, UK

**Keywords:** Health care, Therapeutics

## Abstract

We set out to test whether positive non-verbal behaviours of a virtual coach can enhance people's engagement in automated virtual reality therapy. 120 individuals scoring highly for fear of heights participated. In a two-by-two factor, between-groups, randomised design, participants met a virtual coach that varied in warmth of facial expression (with/without) and affirmative nods (with/without). The virtual coach provided a consultation about treating fear of heights. Participants rated the therapeutic alliance, treatment credibility, and treatment expectancy. Both warm facial expressions (group difference = 7.44 [3.25, 11.62], p = 0.001, ${eta}_{p}^{2}$=0.10) and affirmative nods (group difference = 4.36 [0.21, 8.58], p = 0.040, ${eta}_{p}^{2}$ = 0.04) by the virtual coach independently increased therapeutic alliance. Affirmative nods increased the treatment credibility (group difference = 1.76 [0.34, 3.11], p = 0.015, ${eta}_{p}^{2}$ = 0.05) and expectancy (group difference = 2.28 [0.45, 4.12], p = 0.015, ${eta}_{p}^{2}$ = 0.05) but warm facial expressions did not increase treatment credibility (group difference = 0.64 [− 0.75, 2.02], p = 0.363, ${eta}_{p}^{2}$ = 0.01) or expectancy (group difference = 0.36 [− 1.48, 2.20], p = 0.700, ${eta}_{p}^{2}$ = 0.001). There were no significant interactions between head nods and facial expressions in the occurrence of therapeutic alliance (p = 0.403, ${eta}_{p}^{2}$ = 0.01), credibility (p = 0.072, ${eta}_{p}^{2}$ = 0.03), or expectancy (p = 0.275, ${eta}_{p}^{2}$ = 0.01). Our results demonstrate that in the development of automated VR therapies there is likely to be therapeutic value in detailed consideration of the animations of virtual coaches.

## Introduction

Automated virtual reality (VR) therapy is likely to prove a key approach to scale up the delivery of efficacious psychological treatment for mental health difficulties^1,2^. Without reliance on the relatively scarce resource of trained therapists, but with the opportunity for patients to access help in their own homes via the latest standalone consumer headsets, automated VR therapies offer a route to much greater mental health treatment provision. Virtual coaches—who provide instruction, education, encouragement, and feedback to patients—will thus form a crucial element of VR therapy design. In this paper we set out to test two specific characteristics of the virtual coach’s non-verbal behaviour that could enhance the VR treatment experience. If characteristics of the virtual reality therapist do affect the patient experience—including markers of better treatment outcomes—then there could be a programme of work testing a range of potentially important factors in their realisation.

Therapeutic alliance, a positive relationship between patient and therapist, is a reliable predictor of better mental health treatment outcomes^3,4^, and even affects the efficacy of psychological treatments delivered in digital forms^5–7^. Similarly, patient belief in the credibility of a therapy offered, and expectations of successful outcomes, predict better treatment outcomes^8,9^. Therefore, creating VR coaches that enhance therapeutic alliance and treatment credibility and expectancy could help maximise outcomes from automated VR therapies. Conducting randomized controlled clinical trials to compare treatment outcomes for slight modifications of a virtual coach is not practical, since clinical trials are typically labour and resource intensive studies. Instead, the use of proxy measures for good outcomes, such as therapeutic alliance and treatment credibility and expectancy, provides a pragmatic solution for examining potential treatment effects of variation in a virtual coach.

A growing body of research has focused on the experience of virtual humans in coaching and therapies within non-immersive modalities. For example, an early test from Bickmore and Picard^10^ compared empathic and neutral versions of a virtual exercise advisor presented on a desktop computer. The empathic advisor displayed caring behaviours, such as direct gaze towards the participant and a concerned facial expression when participants felt unwell. Participants perceived more care from the empathic advisor and were more willing to continue the consultation. Likewise, Lawson and Mayer^11^ found that people reported a favourable social connection with a virtual instructor that had a positive voice and body gestures in video coaching. Furthermore, Ter Stal et al.^12^ tested the effects of positive facial expressions and response texts of an online virtual coach, who provided tips on physical activity and healthy nutrition. Results showed that positive text responses from the coach, programmed as responses with a greater number of positive words and longer word count, significantly increased participants' perceived rapport with the coach. However, positive facial expressions did not have a significant effect.

Other studies have looked at virtual humans in mental health digital interventions. DeVault et al.^13^ created a virtual interview program on a desktop computer, where a virtual interviewer assessed people’s distress indicators. They compared two versions of the interviewer—an automated version and a Wizard-of-Oz version in which the human operators triggered the virtual interviewer’s spoken and gestural responses. The results showed that people who experienced the Wizard-of-Oz version reported greater rapport, high system usability, and a strong sense that the virtual human was a good listener. Lisetti et al.^14^ evaluated an intervention for alcohol dependence delivered with an empathic or non-empathic virtual counsellor presented on a computer screen. Adding empathic qualities (e.g. nodding, smiling, head posture mimicry, and eyebrow movement) led to a higher level of trust in the counsellor and a more significant social influence. On the other hand, Ranjbartabar et al.^15^ reported in a study of virtual therapists presented on a computer screen that empathic virtual therapists might not necessarily deliver better emotional outcomes than neutral therapists. Overall, reviews of the use of virtual humans have highlighted the potential benefits of realisation of emotional behaviours in facilitating participant engagement^7,16^.

In studies of virtual humans in VR, research has suggested that characters’ behavioural realism and positive non-verbal communication can enhance their social impact^17–19^. Wu et al.^18^ reported that people perceived stronger social presence and interpersonal attractions when collaborating with a highly expressive virtual human, featuring detailed facial movements and body tracking, compared to a low expressive version. More specifically, non-verbal behaviours such as positive facial expressions with smiles^19^ and responsive nodding^17^ by characters increases perceived friendliness, trust, and bonding in VR social situations. However, relationships with virtual coaches in automated VR therapies for mental health difficulties have not been experimentally examined. Furthermore, the potential influence of participant factors on the experience of a VR coach is unknown. For instance, individuals who are especially mistrustful in everyday life may find it harder to form a therapeutic alliance with a virtual coach^20^, but this has not been tested.

The current study tested the impact of a VR coach’s positive non-verbal behaviours (warmth of facial expression, head nodding) on therapeutic alliance and treatment credibility and expectancy for an acrophobia treatment. Additionally, we tested whether a participant’s level of mistrust may moderate the relationship with a virtual coach. Our primary hypotheses were that the addition of warm facial expressions and affirmative nods would independently enhance the therapeutic alliance and treatment credibility and expectancy. Further, we hypothesised that the combined use of warm facial expressions and affirmative nods would have the strongest positive effect (i.e. there would be a significant interaction).

## Methods

### Experimental design

A balanced two-by-two factorial between-groups experimental design was used. The two factors were warm facial expression (with/without, i.e. neutral face) and affirmative head nods (with/without). Therefore, participants were randomised to one of four virtual coach conditions: (1) neutral face (2) neutral face and affirmative nods (3) warm facial expressions and (4) warm facial expressions and affirmative nods. In all experimental conditions the virtual coach’s facial expression included basic behaviours such as eye blinking and lip syncing. The study was single-blind. Participants were unaware of the study hypotheses or that they were being randomised to interact with one of the different versions of the virtual coach.

We calculated a target sample size for a between-factors ANOVA using G*power 3.1^21^. We specified a medium effect size of partial eta-squared = 0.06 and conventional values of power = 0.80 and α = 0.05. A total of 120 participants (30 per condition) would be needed. A randomization list was created using *Research Randomizer*^22^.

### Participants and recruitment

Participants were primarily recruited via social media advertisements in Oxfordshire. We screened for fear of heights using the *Heights Interpretation Questionnaire (HIQ)*^23^(HIQ score > 29, as used in our trial of automated VR therapy for acrophobia^1^) among the general population. Exclusion criteria were individuals who were (a) under 18 years of age, or who reported (b) having photosensitive epilepsy or a significant visual, hearing or mobility impairment that meant that they would not be able to use VR or (c) taking medication which can cause motion sicknesses.

Ethical approval was received from the University of Oxford Medical Sciences Interdivisional Research Ethics Committee. The study was performed in accordance with relevant guidelines and regulations and written informed consent was obtained from all participants. 120 participants (female = 66, male = 50, non-binary = 4) with a mean age of 44.4 (SD = 16.4) took part in the in-person VR study. Participants had a mean fear of heights score of 43.8 (SD = 10.8). Table 1 presents a summary of participant characteristics.

**Table 1:** Participant characteristics by randomisation group.

	Neutral face (n = 30)	Neutral face with nod (n = 30)	Warm face (n = 30)	Warm face with nod (n = 30)
Age in years, mean (SD), range	40.7 (16.6), 18–70 (range)	48.2 (16.4), 18–72 (range)	41.1 (17), 19–77 (range)	47.8 (14.9), 24–74 (range)
Gender Female (F), male (M), non-binary (NB)	15 F/13 M/2 NB	15 F/15 M	18 F/12 M	18 F/10 M/2 NB
Fear of heights scores (HIQ scores)	43.8 (10.5)	43.7 (10.8)	43.9 (11.2)	43.8 (11.0)
VR experience	1.97 (0.85)	1.63 (0.72)	1.80 (0.96)	1.80 (0.85)

### Apparatus and VR scenario

We used a Windows 10 computer (Intel i7-8700K, Nvidia GeForce GTX 1080Ti, 32 GB RAM) to run the VR scenario and render it on a Meta Quest 2 (Meta, formerly Facebook, 2022) through a wireless connection (Air Link). This VR headset resolution is 1832 × 1920 pixels per eye and was set up at a 90 Hz refresh rate.

**Figure 1:**
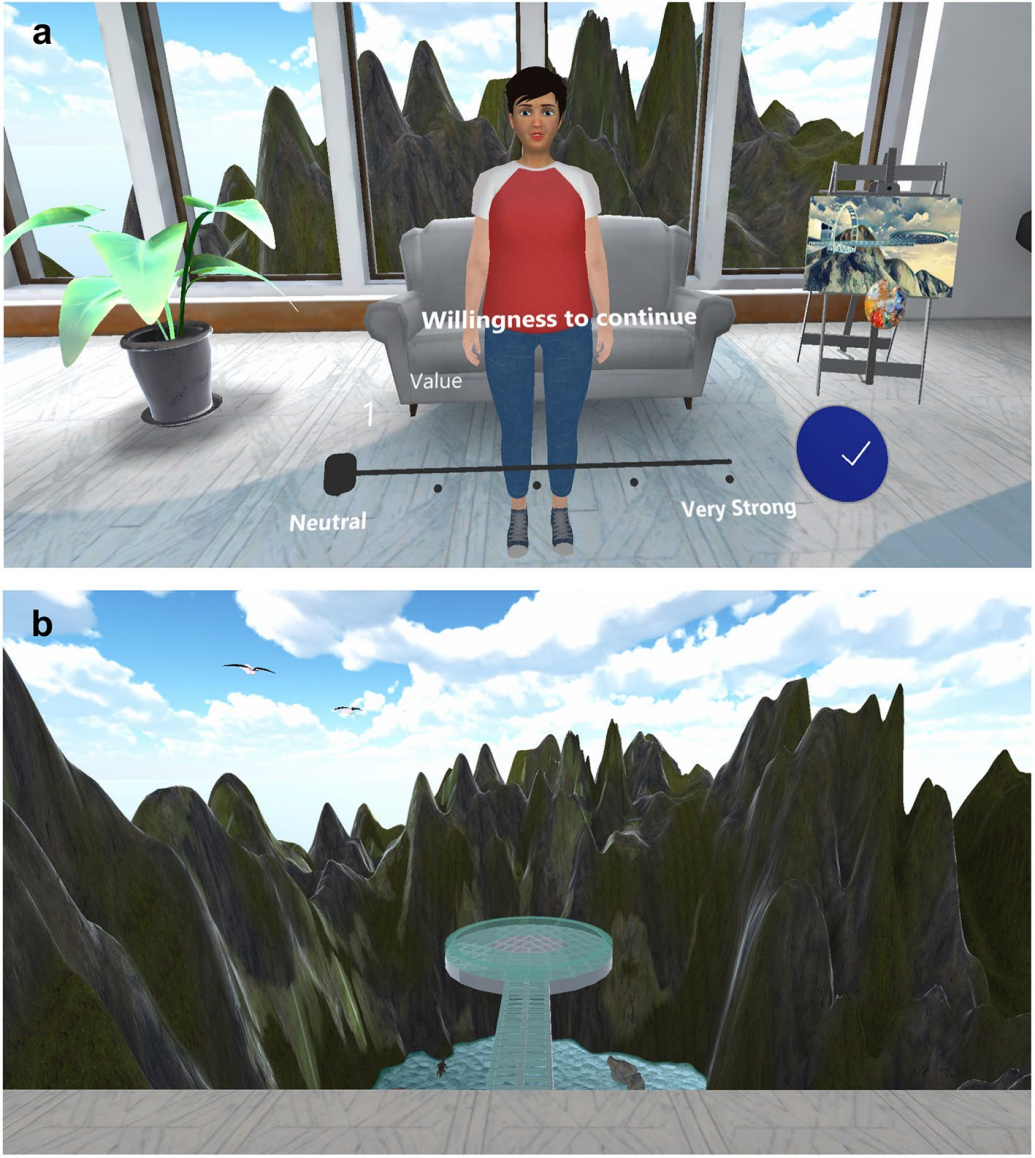
Screenshots of the VR experience. (**a**) Indoor scene: the virtual coach provided an introductory consultation about fear of heights and its treatment. The scene ended with a question about willingness to continue the VR therapy. (**b**) Outdoor scene: participants were instructed to step out on a glass-floor walkway.

We developed the VR experience in Unity game engine, version 2020.3.22. The experience consisted of an indoor scene where participants met the virtual coach for the first time (Fig. 1a) and then they were taken to an outdoor area for a walking task (Fig. 1b). A video of the VR experience is provided as supplementary data.

#### Indoor scene

The indoor scene was a standing experience. Participants faced the virtual coach for an introductory consultation. The consultation script was from our previous VR fear of heights trial^1^. The virtual coach first introduced herself and explained the cognitive approach to understanding fear of heights (e.g. “The reason we’re afraid of heights is because we think something bad is going to happen. And that makes us feel anxious. Then we end up avoiding heights because they feel so scary”). The coach then asked participants questions related to their own fears about heights. Participants answered the questions through a UI interface. They went through this interactive conversation at their own pace, which typically took around 4 min.

#### Outdoor scene

The outdoor scene was also a standing experience in which participants had to walk along an elevated walkway. They started in the middle of a virtual terrace to receive instructions from the virtual coach. The task involved stepping on the walkway, walk until reaching a circular platform, and return to the terrace. The scene concluded once the task was completed or if the participant decided to end it before completion.

We combined the use of motion capture, blend-shape and bone animation to create realistic facial expressions and nods for the virtual coach^24^. A female psychologist was invited as the voice and facial motion actor. The animations were recorded and processed using *Iclone7*^25^ with the *LiveFace* plugin. We ran a pilot test with 12 individuals to verify our character animations of the warm facial expressions and affirmative nods.

### Experimental procedures

**Figure 2:**
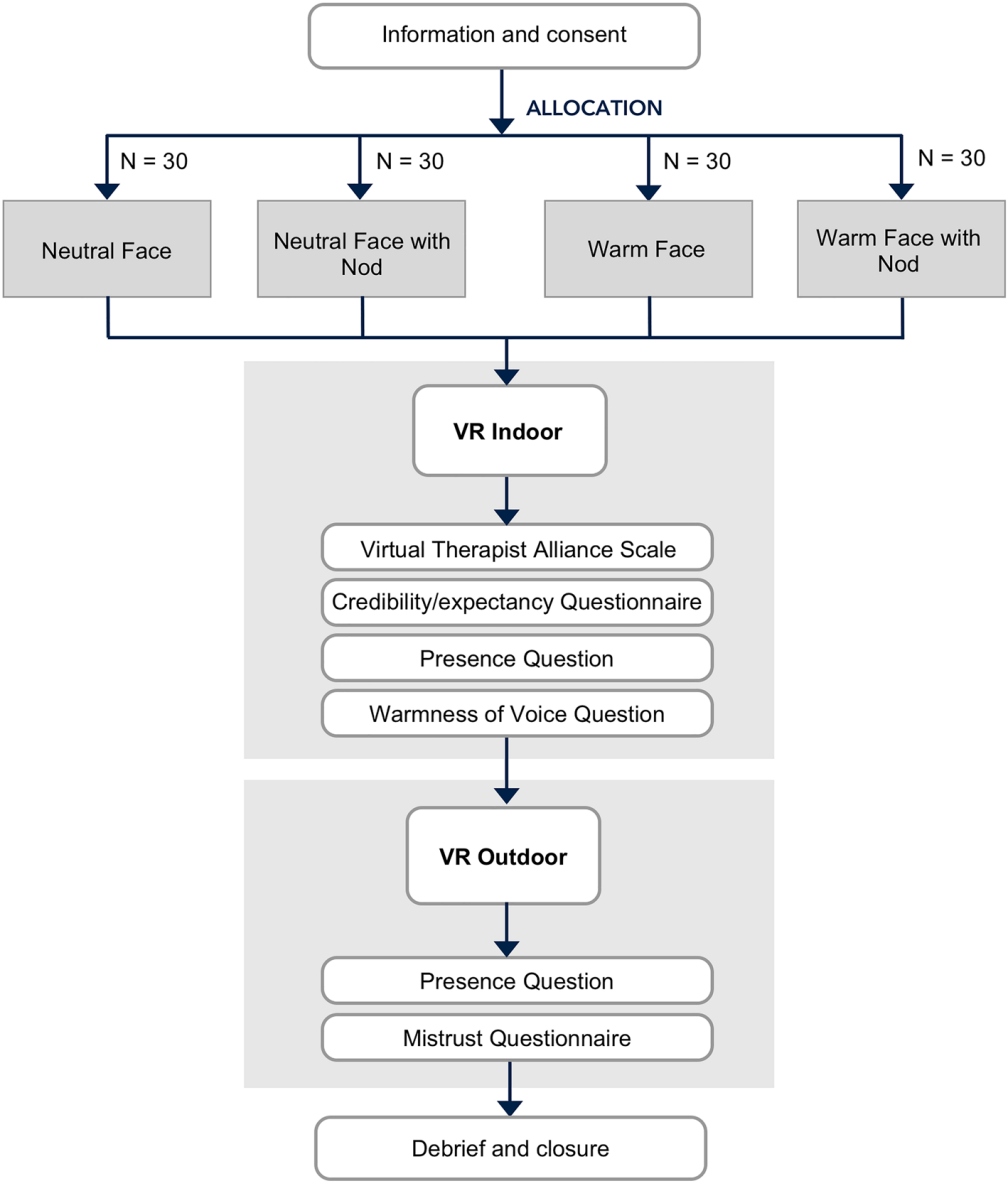
Study procedure.

Participants were invited for a single session at our VR lab. They were informed that they would try the introductory part of a VR therapy for fear of heights. After obtaining written consent to participate in the study, the researcher first demonstrated the use of VR and helped participants fit the VR headset. Later, the researcher selected the parameters for the VR experience according to each participant’s condition group and they experienced the indoor scene. Once that stage ended, participants took the VR headset off and completed the measures of therapeutic alliance, warmness of voice, treatment credibility/expectancy, and presence. The outdoor scene was a virtual heights experience and could elicit anxious feelings for people with fear of heights. The researcher made sure that participants knew beforehand they could stop the VR scene at any time. Participants experienced the outdoor VR scene and then completed the presence and mistrust questionnaires. Finally, they were fully debriefed about the purpose of the study. The entire session lasted approximately 45 min, and participants were reimbursed for their time. Figure 2 shows a summary of the procedure.

### Measures

#### Therapeutic alliance

Alliance with the virtual coach was measured by the *Virtual Therapist Alliance Scale (VTAS)*^6^. It is a 17-item self-report questionnaire describing the perception and relationship with the therapist, such as “The way that the virtual coach communicated was captivating” and “The virtual coach gave me new perspectives on my troubles”. All items are scored from 0 (Do not agree at all) to 4 (Agree completely) using the same response format with total scores ranging from 0 to 68*.* Higher scores reflect a stronger alliance with the virtual coach. The measure had very high internal reliability in this study (Cronbach’s α = 0.94, N = 120).

#### Treatment credibility/expectancy

Treatment credibility and improvement expectancy of the VR fear of heights treatment was measured by the *Credibility/expectancy questionnaire (CEQ)*^26^. It is a six-item questionnaire assessing two factors credibility (three items) and expectancy (three items) separately. Each item is rated in a Likert scale and computed to a score from 1 to 9 (responses to the fourth and the sixth item were linear interpolated from 0 to 100% to 1 to 9), with total scores ranging from 3 to 27 for each factor. Both factors had good internal reliability in this study (credibility: Cronbach’s α = 0.81; expectancy: Cronbach’s α = 0.89).

#### Mistrust

Level of mistrust was measured by *The Revised Green *et al*.*,* Paranoid Thoughts Scale (R-GPTS)*^27^. It is an 18-item scale assessing ideas of persecution, such as “I have been thinking a lot about people avoiding me” and “I was certain people did things in order to annoy me”. All items are scored from 0 (do not agree at all) to 4 (Totally) with total scores ranging from 0 to 72. Higher scores reflect higher levels of mistrust. The measure had very high internal reliability in this study (Cronbach’s α = 0.92).

#### Fear of heights

Fear of heights was measured by the *Heights Interpretation Questionnaire (HIQ)*^23^*.* It is a 16-item self-report questionnaire predicting subjective distress and avoidance of heights. The items assess people’s anxious fears such as the fear of falling or getting hurt, when imagining two height situations (i.e. being on a ladder against a two-story house and on the balcony of a 15th-floor building). The total score ranges from 16 to 80. The measure had good internal reliability in this study (Cronbach’s α = 0.88).

#### Presence

We used a single item from the *Igroup Presence Questionnaire*^28^ to measure sense of presence (“In the computer-generated world I had a sense of ‘being there’”). This measure was simply used to check that both the scenarios led to participants feeling like they were in the virtual environment. The item is scored on a 5-point Likert scale, from 1 (Not at all) to 5 (Very much).

#### Warmness of voice

We used a single item to measure the perceived warmness of voice (“The voice of the virtual coach was warm and friendly”). The item is scored from 0 (Do not agree at all) to 4 (Agree completely).

#### VR behavioural data

We recorded participants’ tracking data (position and rotation) in VR. For the virtual walking task in outdoor VR, we also marked the timestamp and duration corresponding to the key events (step on the walkway, reach the circular platform, back to the terrace).

### Statistical methods

We first checked that the data were suitable for two-way analysis of variance (ANOVA), using Levene’s test for homogeneity of variance and Shapiro–Wilk test of normality (see Supplementary Table S1). The homogeneity of variance was satisfied for all the variables, while the normality assumption was not met for therapeutic alliance, treatment expectancy, presence and warmness of voice. We maintained the original data without transformations due to the robustness of ANOVA to deviations from normality and the sufficient sample size^29^.

To assess the effects of warm facial expressions and affirmative head nods on the therapeutic alliance, treatment credibility and expectancy and other subjective measures, we used a two-way ANOVA test with interaction. The partial eta-squared (${eta}_{p}^{2}$) was computed to measure effect sizes. Tukey's honest significant difference test (Tukey's HSD) was used for multiple pairwise comparisons. All tests for significance were made at the α = 0.05 level. We report the results as mean differences and 95% confidence interval (95% CI) of the difference between conditions.

To assess whether mistrust would moderate the effect of warm facial expressions and affirmative head nods on therapeutic alliance, we used a multiple regression model with the interaction $VirtualCoachAlliance=WarmFace+ AffirmativeNod +AffirmativeNod\times Mistrust+ Mistrust\times WarmFace$. We evaluated the moderating effect based on the significance of the regression coefficient for the interaction term.

Data cleaning and processing was performed using *Python*’s *Pandas* and *NumPy* libraries^30,31^. Analyses were conducted using *R* with *RStudio 1.4*^32^.

## Results

**Figure 3:**
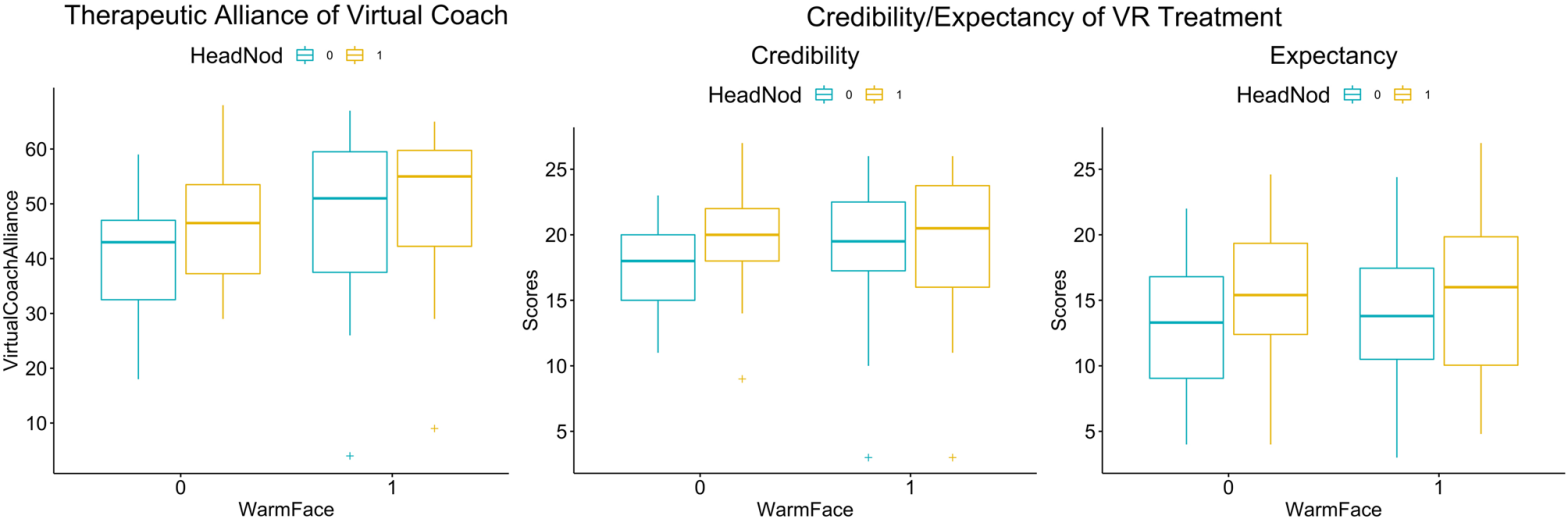
Boxplots of the therapeutic alliance, treatment credibility, and expectancy scores with the two-factor breakdown (0—without, 1—with). Crosses indicate the outlier points, detected at 1.5 times the interquartile range above the upper quartile and below the lower quartile.

Figure 3 shows the raw data box plots for the primary measures of therapeutic alliance, treatment credibility, and expectancy. Descriptive statistics for the measures are shown in Table 2. Apart from two sets of incomplete responses for treatment expectancy items, there were no other missing data. The full details of the analyses can be found in Supplementary Tables S2–S7.

**Table 2:** Descriptive data of measures by randomization group.

Measures	Neutral face	Neutral face with nod	Warm face	Warm face with nod
	Mean (SD)	Mean (SD)	Mean (SD)	Mean (SD)
Therapeutic alliance	40.1 (11.5)	46.2 (10.5)	47.8 (14.9)	50.5 (13.5)
Credibility	17.5 (3.4)	20.1 (4.0)	18.8 (4.9)	19.3 (5.1)
Expectancy	12.8 (5.0)	15.7 (4.9)	14.1 (4.8)	15.1 (6.0)
Presence	7.7 (1.7)	8.2 (1.4)	8.3 (1.2)	8.6 (1.4)
Warmness of the voice	2.8 (1.1)	3.1 (1.1)	3.2 (1.0)	3.5 (0.8)
Mistrust	10.5 (10.3)	9.4 (9.2)	13.8 (11.9)	8.5 (11.5)

### Therapeutic alliance

We removed two extreme outliers (< Q1–3 × IQR) before the two-way ANOVA statistical test. Simple main effects analysis showed that warm facial expressions (group difference = 7.44, 95% CI [3.25, 11.62], F(1, 114) = 12.389, p < 0.001, ${eta}_{p}^{2}$ = 0.10) and affirmative nods (group difference = 4.36, 95% CI [0.21, 8.58], F(1, 114) = 4.318, p = 0.040, ${eta}_{p}^{2}$ = 0.04) led to significant increases in therapeutic alliance. There was no significant interaction between warm facial expressions and affirmative nods (F(1, 114) = 0.705, p = 0.403, ${eta}_{p}^{2}$ = 0.01). Tukey’s HSD Test for multiple comparisons found that therapeutic alliance was significantly greater in the warm face compared to the neutral face condition (p-adj = 0.014) and in the warm face with nod compared to the neutral face condition (p-adj < 0.001).

### Treatment credibility and expectancy

Simple main effects analysis showed that affirmative nods (group difference = 1.76, 95% CI [0.34, 3.11], F(1, 113) = 6.11, p = 0.015, ${eta}_{p}^{2}$ = 0.05) led to significant increases in treatment credibility but that warm facial expressions did not (group difference = 0.64, 95% CI [− 0.75, 2.02], F(1, 113) = 0.833, p = 0.363, ${eta}_{p}^{2}$ = 0.01). There was no statistically significant interaction between warm facial expressions and affirmative nods (F(1, 113) = 3.293, p = 0.072, ${eta}_{p}^{2}$ = 0.03), although there was a trend in the direction of the combination leading to greater credibility ratings. Tukey’s HSD Test for multiple comparisons found that credibility was significantly greater in the neutral face with nod condition compared to the neutral face condition (p-adj = 0.016).

Two participants had incomplete data completion for the expectancy items and were removed from the statistical analysis. Simple main effects analysis showed that affirmative nods (group difference = 2.28, 95% CI [0.45, 4.12], F(1, 114) = 6.055, p = 0.015, ${eta}_{p}^{2}$ = 0.05) led to a significant increase in expectancy but that warm facial expressions did not (group difference = 0.36, 95% CI [− 1.48, 2.20], F(1, 114) = 0.833, p = 0.700, ${eta}_{p}^{2}$ = 0.001). There was no statistically significant interaction between warm facial expressions and affirmative nods (F(1, 114) = 1.202, p = 0.275, ${eta}_{p}^{2}$ = 0.01). Tukey’s HSD Test for multiple comparisons found that mean expectancy was not significantly different between the groups.

### Moderator effect of mistrust

A multiple regression was used to predict therapeutic alliance by the variables of warm facial expression, affirmative nods, and their interaction with mistrust (F(5, 112) = 4.21, p = 0.002, ${R}^{2}$ = 15.83). Both interaction terms WarmFace*Mistrust (p = 0.961) and AffirmativeNods*Mistrust (p = 0.971) were not statistically significant, suggesting mistrust did not moderate the effects.

### Presence

A two-way ANOVA showed that warm facial expressions (group difference = 0.70, 95% CI [0.21, 1.19], F(1, 113) = 8.119, p = 0.005, ${eta}_{p}^{2}$ = 0.07) led to significantly higher levels of presence but that affirmative nods did not (group difference = 0.40, 95% CI [− 0.09, 0.89], F(1, 113) = 2.649, p = 0.106, ${eta}_{p}^{2}$ = 0.02). There was no significant interaction between warm facial expressions and affirmative nods (F(1, 113) = 0.178, p = 0.674, ${eta}_{p}^{2}$ = 0.001). Tukey’s HSD Test for multiple comparisons found that the presence was significantly greater in the warm face with nod compared to the neutral face condition (p-adj = 0.011).

### Warmness of voice

A two-way ANOVA showed that warm facial expressions (group difference = 0.45, 95% CI [0.16, 0.75], F(1, 110) = 9.44, p = 0.003, ${eta}_{p}^{2}$ = 0.08) and affirmative nods (group difference = 0.39, 95% CI [0.09, 0.67], F(1, 110) = 6.54, p = 0.01, ${eta}_{p}^{2}$ = 0.06) led to significantly higher ratings of voice warmness. There was no significant interaction for the combined effects of warm facial expressions and affirmative nods (F(1, 110) = 1.579, p = 0.212, ${eta}_{p}^{2}$ = 0.01). Tukey’s HSD Test for multiple comparisons found that the warmness of the voice was significantly greater in the warm face with nod compared to the neutral face condition (p-adj < 0.001), the warm face condition compared to the neutral face condition (p-adj = 0.017) and the neutral face with nod condition compared to the neutral face condition (p-adj = 0.040).

### Behavioural data

We conducted an exploratory analysis of participants’ walking task performance across the virtual height. Table 3 shows the summary statistics. 82 out of 120 participants (68.33%) completed the task. The average time to move forward and step on to the walkway was 38.0 s (SD = 47.1), and the average duration spent in outdoor VR after the task brief was 113.1 s (SD = 82.0). We also calculated the normalized walking distance based on the horizontal distance of the virtual walkway. Two sets of data were excluded; one participant experienced a VR connection loss and another opted out of the walking task in the outdoor scene. A two-way ANOVA suggested that warm facial expressions (p = 0.187) and affirmative nods (p = 0.374) did not have statistically significant effects on walking distance. Similarly, warm facial expressions and affirmative nods did not have statistically significant effects on the time to step on to the virtual walkway (warm facial expressions: p = 0.356, affirmative nods: p = 0.978) and the time spent in the outdoor scene (warm facial expressions: p = 0.732, affirmative nods: p = 0.511).

**Table 3:** Summary statistics of the VR walking task.

	Task completion	Normalized distance	Duration-StepOnWalkway (s)	Duration-outdoor (s)
	Number (%)	Mean (SD)	Mean (SD)	Mean (SD)
All groups	82 (68.33%)	4.65 (2.73)	38.0 (47.1)	113.1 (82.0)
Neutral face	21 (70.00%)	4.78 (2.68)	36.1 (60.9)	111.5 (93.8)
Neutral face with nod	17 (56.67%)	3.86 (3.00)	50.1 (53.1)	109.5 (71.1)
Warm face	22 (73.33%)	4.96 (2.62)	40.4 (40.1)	124.6 (87.0)
Warm face with nod	22 (73.33%)	4.99 (2.55)	27.5 (28.6)	106.7 (77.2)

## Discussion

Virtual coaches are a key element in automated VR therapies for mental health disorders. We investigated whether introducing positive non-verbal behaviours to the coach increased the therapeutic alliance and treatment credibility and expectancy. Our results partly support our initial hypotheses. We hypothesised that warm facial expressions and affirmative head nods would enhance the therapeutic alliance, treatment credibility, and expectancy, and their combination would have the strongest impact. The results showed that warm facial expressions and affirmative head nods individually affected therapeutic alliance, and the impact of warm facial expressions was more substantial. Additionally, affirmative head nods increased people’s beliefs in both the credibility of the treatment and the expectancy of good outcomes. Although there was no significant interaction between warm facial expressions and affirmative head nods, there was a trend in the direction that the combination led to greater treatment credibility. In essence, how a virtual coach is programmed affects the treatment experience and potentially therapeutic outcomes. In this study we showed that there is likely to be value in implementing facial expressions and positive non-verbal behaviours for the virtual coach.

The primary finding that warm facial expressions and affirmative head nods increase alliance is in line with previous studies of virtual humans outside of the context of VR mental health treatment^11,17,19,33,34^. Similar to the conclusion from Oh et al.^35^ that virtual agents’ facial expressions contribute more than body movements (such as raising of hands and head tilts), the effect size of warm facial expressions of the virtual coach in the current study on the therapeutic alliance was larger than affirmative head nods. Unexpectedly, we did not detect a main effect of warm facial expressions on treatment credibility or expectancy. However, when warm facial expressions were combined with affirmative head nods, there was a trend towards higher credibility ratings. This result might be due to the head nods giving the impression that the therapist was attentively listening and acknowledging participant responses^36^. Such an impression could have then enhanced the potential positive effects of warm facial expressions on treatment credibility when they were displayed simultaneously. Interestingly, positive non-verbal behaviours also led to positive voice perception, which highlights an interplay between perceptions of different sensory traits of virtual humans. In this study, we presented two plausible examples of virtual coach’s behaviours (i.e. facial expressions and head nods) to demonstrate their impact on mental health treatment. Future research could examine other attributes (e.g. visual, auditory, and other non-verbal behaviours such as eye gaze and hand gestures) and their interactive effects.

Our main focus was the effect of characteristics of a coach on established proxies for good therapeutic outcomes. But we also took an exploratory look at potential effects on participants’ behaviours in relation to virtual heights. Approximately one-third of participants did not complete the circuit out to the virtual height and back again. There was no significant difference in the task completion rate, or the distance covered, between the groups allocated to different virtual coach conditions. Since this was the participants' initial exposure to virtual heights, as opposed to the multiple immersions experienced during a full therapy session, we did not make any specific predictions. It would be plausible that the relationship with the virtual coach would make no noticeable difference as patients obtain their first experience of the treatment technique. Indeed, no group differences were detected in whether a person stepped onto the platform or the distance covered.

The study has several limitations. First, we do not know whether the effects of the non-verbal behaviours do translate to better outcomes. This would require a clinical trial to provide evidence. Our view is that using proxies of good outcomes such as therapeutic alliance and treatment credibility is a more sensible testing strategy than conducting multiple clinical trials on small changes to a programme. When such treatments get used at scale then it may be possible to look at outcome effects by programming modifications. Second, we only focused on the virtual coach’s facial expressions and head nods and did not account for factors such as gender, ethnicity, and age of the participants. Previous research indicates that people tend to have stronger bonds with virtual humans with similar characteristics as the person^37^. In the future it is likely that people will be able to customize the appearance, style, and even animations of their virtual coach, which could be studied in relation to therapeutic alliance. Third, we used single-blind testing, with the experimenter being aware of a participant's allocated condition since there was only one experimenter running the study. This design choice may have introduced potential bias during the conduct of the experiment, including the experimenter's greeting style, which could have subsequently influenced participants' subjective ratings. Fourth, mistrust was measured at the end of testing, and this may have affected ratings, and therefore was not actually a true moderator variable. However, there was no clear evidence that mistrust was linked to perceptions of the therapeutic alliance or treatment credibility or expectancy. Finally, the violation of normality in the two-way ANOVA can result in overestimating test significance and increase the chances of Type I error. For example, the p-value of 0.04 for the relationship between nodding and alliance is close to the significance threshold, indicating that a larger sample size will be needed for more robust conclusions.

In this study we investigated the effects of a virtual coach's positive non-verbal behaviours during an automated VR consultation for the treatment of the fear of heights. The inclusion of warm facial expressions and affirmative head nods independently increased therapeutic alliance. Furthermore, affirmative head nods by the virtual coach improved perceptions of treatment credibility and positive outcome expectancy. The findings highlight the potential to enhance the experience and effectiveness of VR therapies through tailored VR character design. While our study focused on the cognitive treatment of fear of heights, further study is needed to examine the degree to which there is generalization to other mental health difficulties and different treatment techniques. The development of VR therapies would benefit from a systematic programme of research of the best attributes of virtual coaches, which may vary depending on the conditions and treatment techniques, and require strong collaborations between clinical staff, people with lived experiences, and software developers.

## Supplementary Information

Supplementary Video 1.

Supplementary Information.

## Data Availability

Deidentified data are available from the corresponding authors on reasonable request and contract with the university.
