# Supplementary material for: A randomised controlled test of emotional attributes of a virtual coach within a virtual reality (VR) mental health treatment
Source: Sci Rep. 2023 Jul 17;13:11517. doi: 10.1038/s41598-023-38499-7 (PMC10352334; doi:10.1038/s41598-023-38499-7)
Supplement: Supplementary file 2 — Supplementary Information. [file 41598_2023_38499_MOESM2_ESM.pdf]

Assumption testing for 2-way ANOVA

Supplementary Table S1: 2-way ANOVA assumption testing results

	Homogeneity of Variance				Shapiro-Wilk Normality Test	
**Measures**	**df1**	**df2**	**F value**	**Pr(>F)**	**w**	**p**
Therapeutic Alliance	3	114	0.527	0.665	0.96	0.001
Treatment Credibility	3	113	0.613	0.608	0.973	0.02
Treatment Expectancy	3	114	0.955	0.417	0.989	0.44
Presence	3	113	2.6	0.056	0.958	0.001
Warmness of Voice	3	110	2.51	0.063	0.921	<0.001

Results for 2-Way ANOVA

<u>Supplementary Table S2: 2-way ANOVA results for alliance</u>

Alliance	Df	Sum Sq	Mean Sq	F value	Pr(>F)	Mean Difference with 95% CI	Eta2 (partial) with 95% CI
WarmFace	1	1631	1631.1	12.389	<0.001 ***	7.437 [3.251,11.622]	0.1 [0.02, 0.21]
HeadNod	1	568	568.5	4.318	0.040 *	4.360 [0.205, 8.575]	0.04 [0.00, 0.13]
WarmFace:HeadNod	1	93	92.8	0.705	0.403		0.006 [0.00, 0.06]
Residuals	114	15009	131.7				

<u>Supplementary Table S3: 2-way ANOVA results for Credibility</u>

Credibility	Df	Sum Sq	Mean Sq	F value	Pr(>F)	Mean Difference with 95% CI	Eta2 (partial) with 95% CI
WarmFace	1	11.9	11.88	0.833	0.363	0.637 [-0.746, 2.021]	0.007 [0.00, 0.07]
HeadNod	1	87.1	87.14	6.110	0.015 *	1.760 [0.343, 3.109]	0.05 [0.00, 0.15]
WarmFace:HeadNod	1	47	46.96	3.293	0.072		0.03 [0.00, 0.11]
Residuals	113	1611.7	14.26				

<u>Supplementary Table S4: 2-way ANOVA results for Expectancy</u>

Expectancy	Df	Sum Sq	Mean Sq	F value	Pr(>F)	Mean Difference with 95% CI	Eta2 (partial) with 95% CI
WarmFace	1	3.8	3.81	0.150	0.700	0.359 [-1.479, 2.198]	0.001 [0.00, 0.04]
HeadNod	1	154	153.96	6.055	0.015 *	2.284 [0.445, 4.124]	0.05 [0.00, 0.15]
WarmFace:HeadNod	1	30.6	30.56	1.202	0.275		0.01 [0.00, 0.08]
Residuals	114	2898.7	25.43				

Supplementary Table S5: 2-way ANOVA results for Warmness of Voice

Warmness of Voice	Df	Sum Sq	Mean Sq	F value	Pr(>F)	Mean Difference with 95% CI	Eta2 (partial) with 95% CI
WarmFace	1	5.93	5.93	9.442	0.003 **	0.454 [0.162, 0.750]	0.08 [0.01, 0.19]
HeadNod	1	4.1	4.104	6.535	0.012 *	0.389 [0.085, 0.674]	0.06 [0.00, 0.16]
WarmFace:HeadNod	1	0.99	0.991	1.579	0.212		0.01 [0.00, 0.09]
Residuals	110	69.08	0.628				

Supplementary Table S6: 2-way ANOVA results for Presence

Presence	Df	Sum Sq	Mean Sq	F value	Pr(>F)	Mean Difference with 95% CI	Eta2 (partial) with 95% CI
WarmFace	1	14.29	14.287	8.119	0.005 **	0.699 [0.213, 1.185]	0.07 [0.01, 0.17]
HeadNod	1	4.66	4.661	2.649	0.106	0.399 [-0.087, 0.885]	0.02 [0.00, 0.10]
WarmFace:HeadNod	1	0.31	0.313	0.178	0.674		0.001 [0.00, 0.05]
Residuals	113	198.86	1.76				

Supplementary Table S7: Multiple pairwise-comparison between the means of groups

Pairwise Condition	Alliance p-adj	Credibility p-adj	Expectancy p-adj	WarmnessOfVoice p-adj	Presence p-adj
WarmFace-NeutralFace	0.013542 *	0.229	0.743	0.016984 *	0.105
NeutralFacewithNod-NeutralFace	0.169	0.0156883 *	0.142	0.0399989 *	0.465
WarmFacewithNod-NeutralFace	0.0007801 ***	0.090	0.330	0.00073 ***	0.0106219 *
NeutralFacewithNod-WarmFace	0.732	0.683	0.662	0.992	0.825
WarmFacewithNod-WarmFace	0.826	0.969	0.903	0.794	0.838
WarmFacewithNod-NeutralFacewithNod	0.236	0.912	0.967	0.632	0.320
